# Incidence and risk factors for recurrence of ampullary adenomas after endoscopic papillectomy: Comparative analysis of familial adenomatous polyposis and sporadic ampullary adenomas in an international multicenter cohort

**DOI:** 10.1111/den.14725

**Published:** 2024-01-10

**Authors:** Achintya D. Singh, Carol A. Burke, Peter V. Draganov, Jay Bapaye, Makoto Nishimura, Saowanee Ngamruengphong, Vladimir Kushnir, Neil Sharma, Vivek Kaul, Aparajita Singh, Amol Bapaye, Debdeep Banerjee, Alexis Bayudan, Mariajose Rojas De Leon, Ritu R. Singh, Shruti Mony, Ashish Gandhi, Thomas Hollander, Krystle Bittner, Jacques Beauvais, Ruishen Lyu, David Liska, Tyler Stevens, Matthew Walsh, Amit Bhatt

**Affiliations:** ^1^ Department of Internal Medicine Cleveland Clinic Cleveland OH USA; ^2^ Department of Gastroenterology and Hepatology Cleveland Clinic Cleveland OH USA; ^3^ Department of Quantitative Health Sciences Cleveland Clinic Cleveland OH USA; ^4^ Department of General Surgery Cleveland Clinic Cleveland OH USA; ^5^ Department of Colorectal Surgery, Digestive Disease and Surgery Institute Cleveland Clinic Cleveland OH USA; ^6^ Sanford R. Weiss MD Center for Hereditary Colorectal Neoplasia, Digestive Disease and Surgery Institute Cleveland Clinic Cleveland OH USA; ^7^ Department of Gastroenterology and Hepatology, MetroHealth Medical Center Case Western Reserve University Cleveland OH USA; ^8^ Division of Gastroenterology, Hepatology and Nutrition University of Florida Gainesville FL USA; ^9^ Internal Medicine Rochester General Hospital Rochester NY USA; ^10^ Division of Gastroenterology, Hepatology and Nutrition Service Memorial Sloan Kettering Cancer Center New York NY USA; ^11^ Division of Gastroenterology and Hepatology University of Rochester Medical Center Rochester NY USA; ^12^ Department of Gastroenterology and Hepatology The Johns Hopkins Hospital Baltimore MD USA; ^13^ Division of Gastroenterology Washington University School of Medicine St. Louis MO USA; ^14^ Division of Interventional Oncology and Surgical Endoscopy (IOSE) Parkview Cancer Institute Fort Wayne IN USA; ^15^ Department of Gastroenterology University of California San Francisco CA USA; ^16^ Department of Gastroenterology and Hepatology University of Oklahoma Norman OK USA; ^17^ Shivanand Desai Center for Digestive Disorders Deenanath Mangeshkar Hospital and Research Center Pune India

**Keywords:** ampullary adenoma, ampullary cancer, endoscopic papillectomy, familial adenomatous polyposis, recurrence

## Abstract

**Objectives:**

Endoscopic papillectomy (EP) is a minimally invasive therapy for the management of ampullary adenomas (AA). We conducted this multicenter study to assess the incidence of and factors related to the recurrence of AA after EP in patients with familial adenomatous polyposis (FAP) compared to sporadic AA.

**Methods:**

We included patients who underwent EP for AA at 10 tertiary hospitals. Adenomatous tissue at the resection site at the time of surveillance endoscopies was considered recurrent disease.

**Results:**

In all, 257 patients, 100 (38.9%) with FAP and 157 (61%) patients with sporadic AA, were included. Over a median of 31 (range, 11–61) months, recurrence occurred in 48/100 (48%) of patients with FAP and 58/157 (36.9%) with sporadic AA (*P* = 0.07). Two (2%) FAP patients and 10 (6.3%) patients with sporadic AA underwent surgery for recurrence. On multivariable regression analysis, the recurrence in FAP was higher than in sporadic patients after the first year of follow‐up. AA size (hazard ratio [HR] 1.03, 95% confidence interval [CI] 1.001, 1.056), periampullary extension (HR 2.5, 95% CI 1.5, 4.01), and biliary duct dilation (HR 2.04, 95% CI 1.2, 3.4) increased the risk, while en bloc resection (HR 0.6, 95% CI 0.41, 0.9) decreased the risk of recurrence.

**Conclusion:**

Recurrence rates are high after EP. Most recurrences in sporadic patients occur within the first year of follow‐up, but after the first year of follow‐up in patients with FAP. Recurrences are higher with larger adenomas, biliary duct dilation, and periampullary extensions, and may be mitigated by en bloc resection. These factors should be considered in decision‐making with the patients.

## INTRODUCTION

Ampullary adenomas (AA) are premalignant lesions seen in 29–72% patients with familial adenomatous polyposis (FAP), but is also seen in the general population.^1^, ^2^, ^3^ We previously found that in patients with FAP and AA, 20% had clinically significant progression requiring an intervention.^4^

Endoscopic papillectomy (EP) is a minimally invasive resection technique for AA. In patients with FAP, the American Society for Gastrointestinal Endoscopy (ASGE) recommends consideration of EP for AA ≥1 cm, advanced histology, high‐grade dysplasia (HGD) and/or in patients with obstructive symptoms including abnormal liver function tests or pancreatitis.^3^, ^5^ The European Society for Gastrointestinal Endoscopy (ESGE) suggests that AA >1 cm in size that are showing excessive growth or suspicion of invasive growth should undergo evaluation in a multidisciplinary setting for further resection.^6^ Despite its effectiveness, EP is associated with high recurrence rates, ranging from 11.8% in sporadic cases to 52.2% in patients with FAP (Table S1).^7^, ^8^, ^9^, ^10^, ^11^, ^12^ Unfortunately, most studies of EP in patients with FAP were limited by a small sample size, single‐center studies, and short follow‐up time.^3^, ^5^, ^13^

We conducted the present international, multicenter retrospective study to estimate the recurrence of AA after EP in patients with FAP compared to sporadic AA and assess the demographic, clinical, histologic, and procedural factors associated with recurrence.

## METHODS

### Study design

This multicenter, retrospective cohort study was performed in patients with AA undergoing EP across 10 tertiary referral centers in the United States (*n* = 8), India (*n* = 1), and Brazil (*n* = 1) between the January 1, 2000 to March 1, 2022. Institutional Review Board approval was obtained from each of the participating centers. Study data were collected and managed using the REDCap electronic data capture system hosted at the Cleveland Clinic.^14^, ^15^

### Patient selection

Patients with AA treated by EP were included. Patients with ampullary cancer, prior resection of AA, nonadenomatous pathology, no post‐EP surveillance, or those with residual AA after EP AA that could not be completely treated were excluded.

### Procedures

All procedures were performed by interventional endoscopists. The technique used was at the discretion of the endoscopist. The size of AA was estimated using an open snare of known size or visually estimated by the endoscopist. The resected specimens were assessed by expert gastrointestinal pathologists. The tissue histology was assessed as per the Vienna classification.^16^

### Surveillance protocol

If a pancreatic stent was placed, a follow‐up endoscopy for stent removal or a plain abdominal X‐ray was performed in 1–4 weeks. If a stent was present, an upper endoscopy (esophagogastroduodenoscopy [EGD]) was performed for stent removal. The first follow‐up EGD to assess for recurrent tissue was usually performed within 4–6 months of the initial procedure. In patients with FAP, further surveillance intervals were based on Spigelman stage (SS) of duodenal polyposis. Patients with sporadic AA also underwent endoscopic surveillance and the intervals were tailored as per the discretion of the treating physicians.^5^

### Definitions

Recurrence was defined as a histologically diagnosed adenomatous lesion on the ampulla or the periampullary region after EP. The resection was recorded as en bloc when the target specimen was removed as a single piece with no endoscopically visible residual tissue. Those en bloc resections with negative margins were regarded as “R0” resections and those with positive margins were considered “R1” resections. Patients with R1 resections had surveillance endoscopies at shorter intervals. The resection was recorded as piecemeal if the specimen was resected in multiple pieces and no endoscopically visible tissue was seen at completion of the procedure.

Procedure‐related adverse events (AEs) were defined as per the established lexicon by the ASGE.^17^ Adverse events occurring up to 14 days after the procedure were considered early AE, while those after 14 days were considered late AE. The follow‐up of patients was censored at the time of the first recurrence.

### Primary and secondary aims

The primary outcome of the study was to assess the recurrence of AA after EP in patients with FAP compared to patients with sporadic AA. The secondary outcome was to assess the clinical, histological, and technical factors associated with recurrence of AA after EP.

### Statistical analysis

To assess the recurrence between two groups: FAP‐associated and sporadic patients, a propensity score (PS)‐weighted Cox proportional hazards (PH) regression model was performed. Propensity score weights were assessed by a logistic regression model with race, age, sex, clinical symptoms, preprocedural imaging findings, AA size, histology, type of resection, electrocautery settings, adjuvant ablation therapy, and medical centers as independent variables and AA types as dependent variables. Patients with extreme weights were trimmed for the weighted Cox PH models. Confounders with a standardized mean difference greater than 0.1 after weighting were included in the final model to adjust for imbalance. In the model, time to recurrence was stratified as “before 12 months” and “at or after 12 months” and etiology of AA, to assess the difference in recurrence between FAP and sporadic AA before and after 12 months.

Univariate analysis and multivariate analysis with Cox PH regression models with cluster and medical center were also performed. Stepwise variable selection based on the Akaike information criterion was used to choose the final model. Kaplan–Meier recurrence‐free curve with 95% confidence intervals (CIs) after PS weighting was constructed and presented with a log‐rank test result. Analysis was performed using R (version 3.6.2; Vienna, Austria) and SAS (version 9.4; Cary, NC, USA) software and a *P*‐value <0.05 was considered statistically significant.

## RESULTS

**Figure 1:**
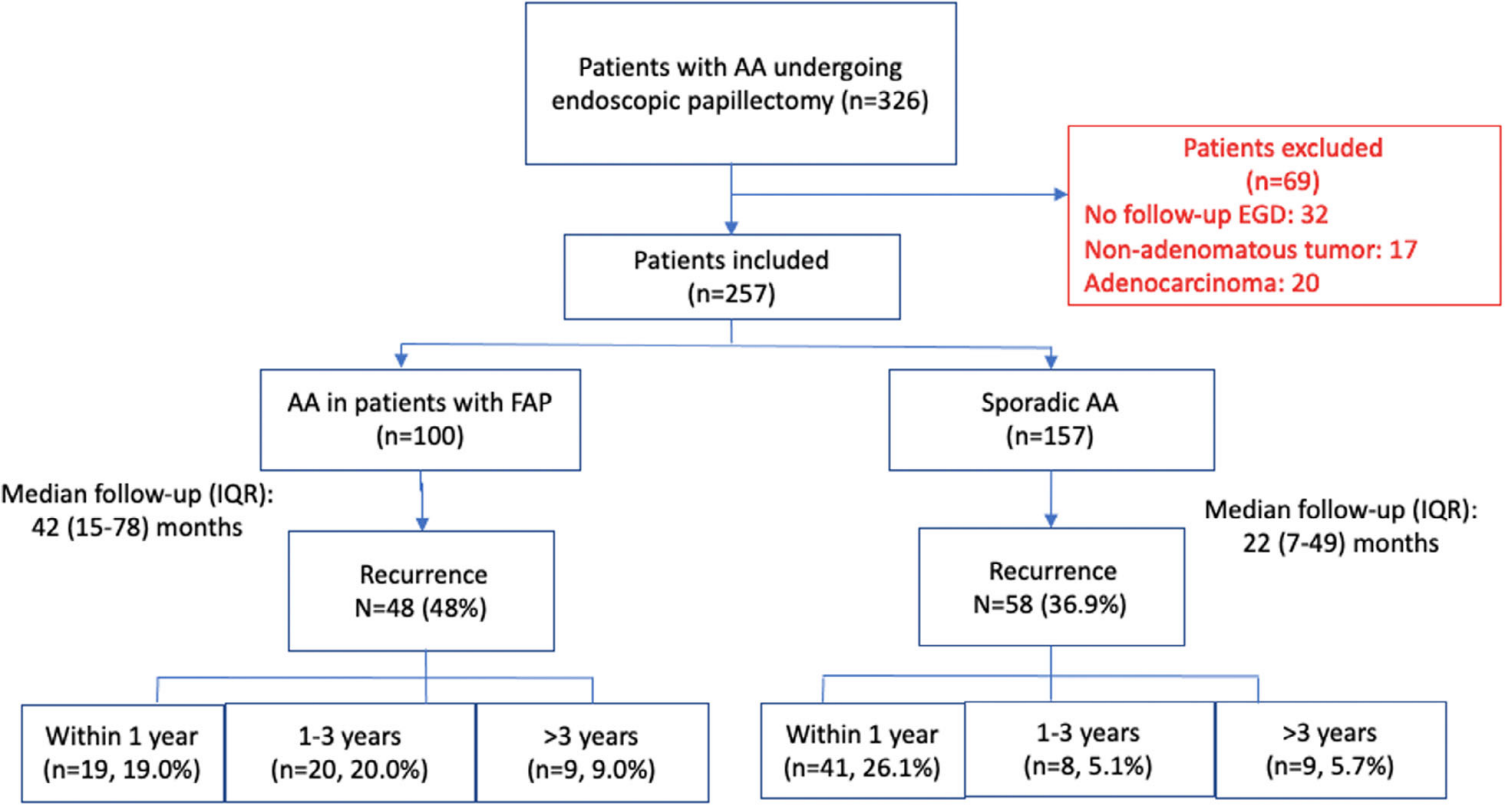
Study flow diagram. AA, ampullary adenomas; EGD, esophagogastroduodenoscopy; FAP, familial adenomatous polyposis; IQR, interquartile range.

After screening 326 patients with AA who underwent EP, 257 patients (comprised of 100 with FAP and 157 patients with sporadic AA) were included. We excluded 69 patients (20 with ampullary cancer, 17 with nonadenomatous pathology, 32 with no surveillance endoscopy) (Fig. 1).

The baseline characteristics of the patients can be found in Table 1. The median patients in the sporadic group were older (*P* < 0.01), had a higher prevalence of women (*P* = 0.07) and Caucasian ethnicity (*P* = 0.03), and more frequently had symptoms referrable to the AA (*P* < 0.01) than FAP patients. The median size of AA in the sporadic group was larger compared to the FAP group (*P* < 0.01). Tubular adenoma (TA) was the most common underlying histology in both groups. There were more HGD in sporadic patients (19.7% vs. 4.3%) compared to FAP (*P* < 0.01). Periampullary extension of the AA was similar in the sporadic (16.9%) and FAP (23.9%) population (*P* = 0.22). The details of the EP procedures can be found in Table 2. Both the groups had similar use of submucosal injection (*P* = 0.13), electrocautery settings (*P* = 0.45), and rates of en bloc resection (*P* = 0.30).

**Table 1:** Baseline characteristics of study population according to etiology of ampullary adenoma (AA)

Characteristic	Total (*N* = 257)	Sporadic (*N* = 157)	Familial (*N* = 100)	*P*‐value
Age, median (IQR)	60.0 (49.0, 72.0)	67.0 (59.0, 74.0)	44.0 (34.5, 55.5)	<0.01
Sex, female, *n* (%)	115 (44.9)	77 (49.4)	38 (38.0)	0.07
Race, Caucasian, *n* (%)	199 (78.0)	115 (73.7)	84 (84.8)	0.03
AA symptomatic, *n* (%)	63 (24.8)	50 (32.3)	13 (13.1)	<0.01
Preprocedural imaging, *n* (%)				
No abnormalities	77 (30.0)	38 (24.2)	39 (39.0)	0.01
Biliary intraductal extension	9 (3.5)	6 (3.8)	3 (3.0)	0.99
Biliary duct dilation	66 (25.7)	59 (37.6)	7 (7.0)	<0.01
Pancreatic duct dilation	29 (11.3)	24 (15.3)	5 (5.0)	0.01
Medical center, *n* (%)				0.03
Cleveland Clinic	67 (26.1)	31 (19.7)	36 (36.0)	–
University of Florida	43 (16.7)	32 (20.4)	11 (11.0)	–
Johns Hopkins	34 (13.2)	20 (12.7)	14 (14.0)	–
MSKCC	23 (8.9)	14 (8.9)	9 (9.0)	–
Other	90 (35.0)	60 (38.2)	30 (30.0)	–
Follow‐up period, *n* (%)				0.02
<1 year	113 (44.0)	81 (51.6)	32 (32.0)	–
1–3 years	77 (30.0)	41 (26.1)	36 (36.0)	–
3–5 years	42 (16.3)	22 (14.0)	20 (20.0)	–
>5 years	25 (9.7)	13 (8.3)	12 (12.0)	–

IQR, interquartile range; MSKCC, Memorial Sloan Kettering Cancer Center.

**Table 2:** Characteristics of ampullary adenomas (AA) and endoscopic papillectomy technique

Characteristic	Total (*N* = 257)	Sporadic (*N* = 157)	Familial (*N* = 100)	*P*‐value
AA size (mm), *n* (%)				<0.01
<10	29 (13.1)	8 (5.9)	21 (24.4)	–
10–30	174 (78.7)	112 (83.0)	62 (72.1)	–
>30	18 (8.1)	15 (11.1)	3 (3.5)	–
AA size (mm)	15 (10.0, 20.0)	18 (12, 25.0)	12.0 (10.0, 20.0)	–
Postpapillectomy histology, *n* (%)				<0.01
TA‐LGD	180 (70.0)	96 (61.1)	84 (84.0)	–
TVA‐LGD/VA‐LGD	36 (14.0)	27 (17.2)	9 (9.0)	–
HGD	35 (13.6)	31 (19.7)	4 (4.0)	–
Periampullary extension, *n* (%)	43 (20.9)	28 (23.9)	15 (16.9)	0.22
Papillectomy technique, *n* (%)				0.13
EP with submucosal injection	135 (52.9)	88 (56.8)	47 (47.0)	–
EP alone	120 (47.1)	67 (43.2)	53 (53.0)	–
Electrocautery settings, *n* (%)				0.45
Forced coagulation current	21 (8.6)	15 (10.1)	6 (6.4)	–
Blended current (Endocut)	114 (46.9)	66 (44.3)	48 (51.1)	–
Unknown	108 (44.4)	68 (45.6)	40 (42.6)	–
Type of resection, *n* (%)				0.30
En bloc	123 (49.0)	71 (46.4)	52 (53.1)	–
Negative margins (R0)	60 (48.8)	39 (54.9)	21 (40.4)	–
Positive margins (R1)	23 (18.6)	14 (19.7)	9 (17.3)	–
Unknown	40 (32.5)	18 (25.4)	22 (42.3)	–
Piecemeal	128 (51.0)	82 (53.6)	46 (46.9)	–
Adjuvant ablation therapy, *n* (%)				
APC	82 (31.9)	55 (35.0)	27 (27.0)	0.18
Snare tip	17 (6.6)	8 (5.1)	9 (9.0)	0.22

APC, argon plasma coagulation; EP, endoscopic papillectomy; HGD, high‐grade dysplasia; LGD, low‐grade dysplasia; TA, tubular adenoma; TVA, tubulovillous adenoma; VA, villous adenoma.

### Primary aim: Recurrence after EP in patients with FAP compared to sporadic AA

Of the 257 patients, 106 (41.2%) patients had recurrence at a median follow‐up of 9 (interquartile range [IQR] 2–24) months. AA recurrence was noted in 48/100 (48%) of patients with FAP and in 58/157 (36.9%) sporadic patients (*P* = 0.82).

**Figure 2:**
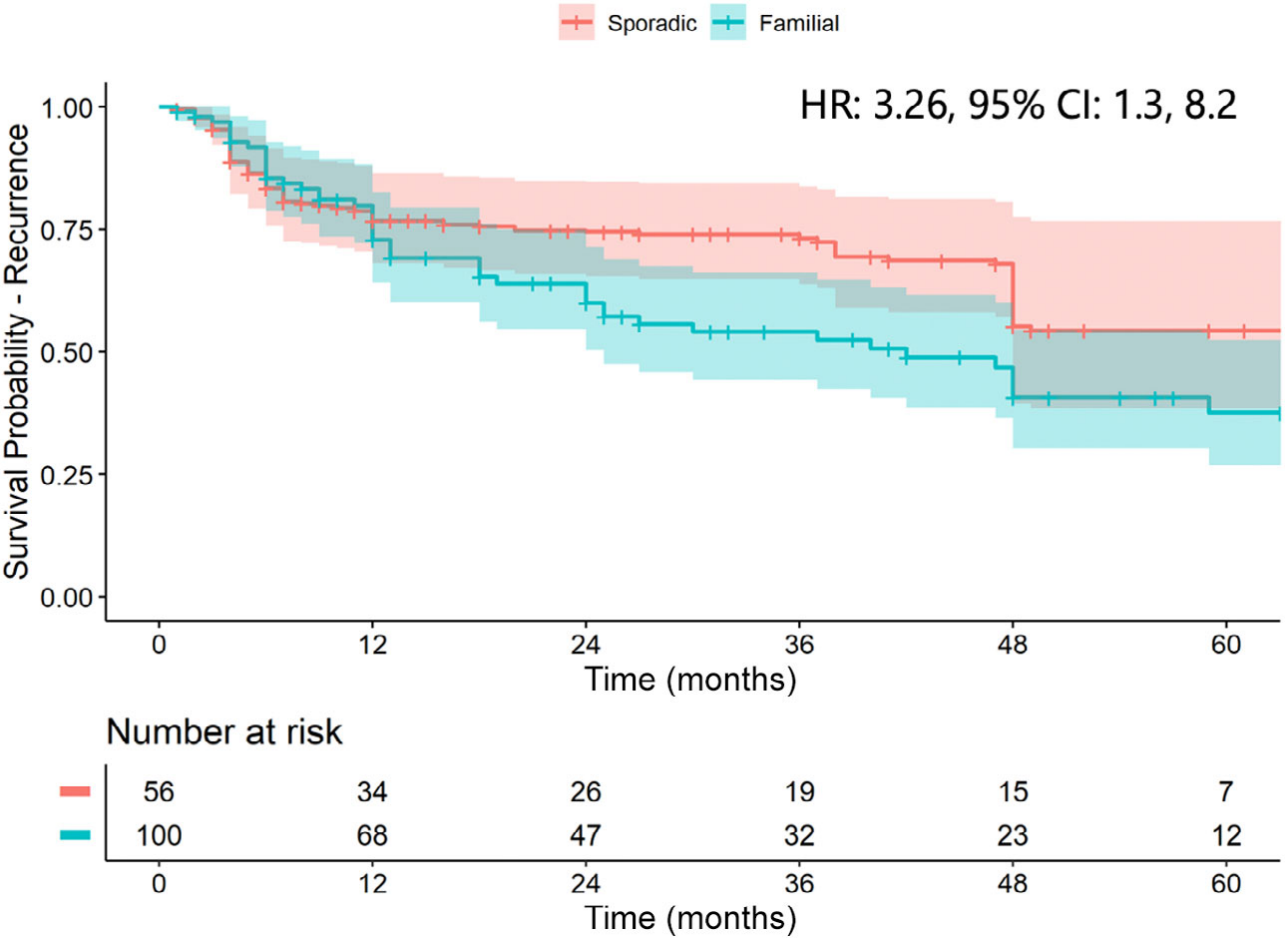
Kaplan–Meier curve for recurrence of ampullary adenomas after propensity score weighting. CI, confidence interval; HR, hazard ratio.

The cumulative recurrence was similar between groups (hazard ratio [HR] 0.95, 95% CI 0.65, 1.40) (Fig. S1). The differences in the baseline characteristics, between the FAP and sporadic group including size and histology of AA, medical center, and follow‐up period were adjusted using a propensity‐weighted model (Fig. S2). There was a significant increase in the recurrence of AA in patients with FAP compared to the sporadic population after the first year of follow‐up (HR 3.26, 95% CI 1.3, 8.2) (Fig. 2). There was no difference in the recurrence rates of various centers involved in the study.

### Secondary aim: Factors associated with recurrence of the ampullary adenomas

The unadjusted regression analysis is detailed in Table S2. On multivariable regression analysis, the risk was higher in patients with FAP after the first 12 months of follow‐up (HR 2.2; 95% CI 1.1, 4.5), biliary duct dilation on the pre‐resection imaging studies (HR 2.04, 95% CI 1.2, 3.4), larger AA size (HR 1.03, 95% CI 1.001, 1.06), and periampullary extension of the AA (HR 2.5, 95% CI 1.5, 4.0). En bloc compared to piecemeal resection (HR 0.63, 95% CI 0.41, 0.9) decreased the risk of recurrence (Table 3).

**Table 3:** Multivariable Cox proportional hazards regression model for factors associated with recurrence of ampullary adenomas (AA)

Factors	Hazard ratio	95% confidence interval	*P*‐value
AA type: Familial vs. sporadic (≤12 months)	1.02	0.60, 1.73	0.94
AA type: Familial vs. sporadic (>12 months)	2.23	1.11, 4.48	0.02
Preprocedural imaging findings: Normal vs. abnormal	0.61	0.36, 1.046	0.07
Preprocedural imaging findings: Biliary duct dilation: Yes vs. no	2.04	1.20, 3.44	0.01
AA size at resection (mm)	1.03	1.00, 1.06	0.04
AA size (size in mm): 10–30 vs. <10	1.64	0.79, 3.38	0.18
AA size (size in mm): >30 vs. <10	0.66	0.19, 2.25	0.51
Periampullary involvement: Yes vs. no	2.48	1.53, 4.01	<0.01
Electrocautery settings: Coagulation vs. Endocut	1.83	0.87, 3.85	0.11
Resection: En bloc vs. piecemeal	0.63	0.41, 0.97	0.04
Adjuvant ablation therapy of the AA borders: Yes vs. no	0.64	0.37, 1.10	0.11

AA, Ampullary adenoma.

### Management of recurrence: Patients with FAP

Of the 48 patients with recurrence in the FAP group, the histologic recurrence in 41 (85.4%) was TA, and in seven (14.5%) patients, it was tubulovillous adenoma (TVA) or villous adenoma (VA). No patients had HGD or cancer on the first recurrence (Table S3).

**Figure 3:**
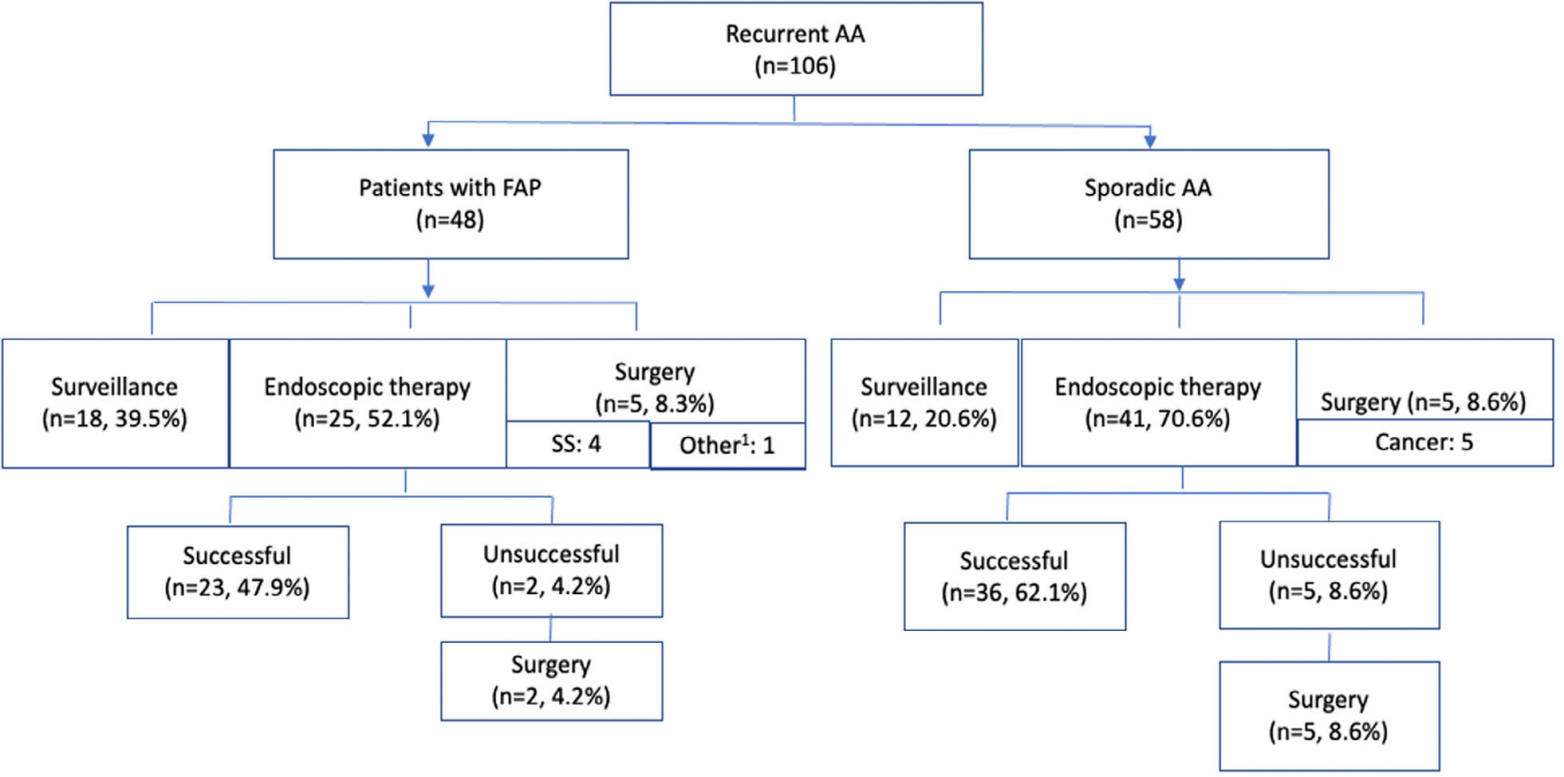
Management of recurrent ampullary adenomas (AAs) in patients with familial adenomatous polyposis (FAP) and in sporadic patients. ^1^Desmoid tumor. SS, Spigelman stage.

Repeat EP was performed in 25/48 (73%) FAP patients, at a median of 13 months (range, 3–53 months). Five patients required surgery due to nonampullary reasons (four patients had advanced SS, one developed intestinal obstruction due to desmoid tumor), and endoscopic surveillance was performed in the remaining patients (Fig. 3). Repeat EP was technically successful in 23/25 (92%) of patients. Two patients with unsuccessful resection underwent surgery, at a median of 39 months (range, 9–118 months). On follow‐up after primary recurrence, one patient developed ampullary cancer 22 months after the initial resection and underwent pylorus‐preserving pancreaticoduodenectomy.

### Management of recurrence: Patients with sporadic ampullary adenomas

In the sporadic group, 58/157 (36.9%) patients had recurrence of AA after a median follow‐up of 6 months (IQR 4, 12 months). The postresection surveillance histology was TA in 39 (67.2%), TVA or VA in seven (12.0%), six (10.3%) had HGD, and six (10.3%) developed ampullary cancer. The management of these patients included repeat EP in 41/58 (70.6%) patients, endoscopic surveillance in 12/58 (20.6%), and surgery in 5/58 (8.6%) patients. Repeat EP was technically successful in 36/41 (88%) patients, but complete resection was not achieved in 5/41 (12%) patients. Five patients with ampullary cancer subsequently underwent surgical resection, while one patient died before surgery (Fig. 3).

### Adverse events: In patients with FAP

Adverse events occurred in 21/100 (21%) patients in the FAP group. Postprocedure pancreatitis was reported in 11 (11%) patients, bleeding in seven (7%) patients, and two (2%) patients had duodenal perforation during the EP. On follow‐up, papillary stenosis was reported in two patients.

### Adverse events: In the sporadic population

Adverse events were reported in 45/157 (28.6%) in the sporadic group. Postprocedure pancreatitis was reported in 17 (10.8%) patients, bleeding occurred in 21 (13.4%) patients, and duodenal perforation was reported in one (0.6%) patient. On follow‐up, four (2.5%) patients developed papillary stenosis, and cholangitis occurred in three of these patients.

## DISCUSSION

In our study, a high recurrence of AA was seen in both the populations, and most of the recurrences occurred in the first year of follow‐up. After the first year of follow‐up, the rate of recurrence increased in patients with FAP. The baseline size of AA was larger, and the histology of AA was more advanced in patients with sporadic AA compared to patients with FAP. The size of AA, periampullary extension of AA, and biliary duct dilation on pre‐resection imaging were associated with an increased risk of recurrence, while en bloc resection of AA reduced the risk of recurrence. To the best of our knowledge, this is the largest study to date comparing the outcomes of EP for AA in patients with FAP compared to those with sporadic AA.

The present literature regarding the recurrence of AA is predominantly derived from patients with sporadic AA. A recent multicenter study of 154 patients, including 43 (27.9%) patients with FAP, found a recurrence of 21.6% in patients with FAP that was not significantly different from those with sporadic AA (13.6%).^10^ Another large retrospective series of 253 patients including only 16 (6.3%) with FAP found a recurrence of 38.4% in FAP compared to 9.5% in the sporadic group.^11^ Studies including only FAP patients have reported a much higher incidence of recurrence ranging from 10% to 58% (Table S1). Also, most studies have found that the risk of recurrence after EP is highest in the first year of follow‐up.^18^, ^19^ We found high recurrence rates of AA after EP in both patients with FAP and those with sporadic AA. This is similar to a recent propensity‐matched study that found a recurrence of 21% in FAP patients compared to 16% in the sporadic population.^20^ While the recurrence rates in the FAP group was similar to prior studies, the recurrence in the sporadic group was higher than previously reported (Table S1). This could be due to differences in the definition of recurrence in the published studies. We found a high number of reported recurrences during the first surveillance endoscopy, and this could represent residual disease. Clinically, residual and recurrent disease result in the same outcome of requiring further endoscopic or surgical intervention. There was no difference based on the various centers involved in the study that could influence the recurrence rates.

The AA in patients with FAP were predominantly detected during surveillance EGD and was likely the reason for the differences in size and histology compared to the sporadic group. The higher recurrence of AA in FAP patients after the first year may be due to the underlying *APC* mutation and their inherent risk of developing polyps.^4^ The similar risk of recurrence between the groups during the first year of follow‐up emphasizes the need for assiduous surveillance in the first year after EP. Also, most of the recurrences were managed endoscopically in both groups. This highlights the importance of close monitoring for recurrences in these patients. This is in agreement with the recent consensus of international experts that recommends patients with AA should have a repeat EGD within 6 months of resection with another within 1 year.^21^ In patients with AA harboring HGD, repeat EGD should be within 3 months of resection and a second follow‐up at 6 months. In all patients, surveillance should be continued for at least 5 years after resection. The ASGE guidelines recommend close surveillance after EP but specific intervals are not endorsed.^5^

Some factors have been shown to affect the risk of AA recurrence after EP. In a study of 102 patients, AA <2 cm and the absence of any ductal dilation were associated with a decreased risk of recurrence within the first year of follow‐up.^19^ Similarly, we found that the absence of biliary dilation and small AA size were associated with a decreased risk of recurrence. Not surprisingly, we found en bloc resection decreased the risk of recurrence. This finding and the absence of intraductal extension of AA in FAP has been corroborated in another study.^11^

The results of our study emphasizes the importance of appropriate patient selection and informs clinical practice regarding the risk of AEs and recurrence after EP. We previously found that only 28.6% patients with FAP and AA have a clinically significant progression over a median follow‐up of about 8 years.^4^ Asymptomatic patients with FAP and small AA (<1 cm), can undergo surveillance endoscopies based on their SS without resection. In asymptomatic FAP patients with advanced adenomas (size >1 mm or villous features), surveillance endoscopies should be repeated within 1 year. Patients with FAP and HGD should undergo EP. The risks and benefits of EP should be appropriately weighed before the procedure and a shared decision should be made.

In both FAP and sporadic patients, if EP is performed, patients with large AA, periampullary extension, or associated biliary duct dilation have a higher risk of progression and may benefit from closer surveillance endoscopies after resection. En bloc resection is the optimal resection method for these tumors. Also, patients with FAP and high SS can be considered for duodenectomy. The role of devices like the wire‐traction device to enhance mucosal exposure to assess AA needs to be explored in the future.^22^

Our study is limited by its retrospective nature and performance at tertiary care centers. To ensure generalizability of the data, we used standard published definitions for reporting AE.^5^, ^17^ All the study outcomes were predefined to avoid any measurement bias. The follow‐up period was longer in patients with FAP than in the sporadic group, since they were in surveillance programs for duodenal polyposis. Also, information regarding positivity of vertical/horizontal margins was not collected uniformly across all centers, and its impact on recurrence of AA could not be assessed. The strengths of our study include comparing outcomes in the largest number of patients with FAP with a similar number of patients with sporadic AA. Additionally, our patients were drawn from three countries with very different demographics. This provides generalizability to our study results. Future large‐scale studies looking at the appropriate interval for surveillance endoscopy after EP are needed. The impact of positive margins after resection of ampullary tumors on the recurrence rates needs to be explored in future studies.

## CONCLUSION

Endoscopic papillectomy is associated with high recurrence rates in FAP and sporadic patients. While most recurrences in sporadic patients occur within the first year of follow‐up, recurrence in patients with FAP occurs within 1–3 years of follow‐up. We confirmed the recurrence of larger adenomas, those associated with biliary duct dilation, or with periampullary extension and recurrence may be mitigated by en bloc resection. Most recurrences can be managed with additional endoscopic treatment. Due to a high rate of AEs, lesion recurrence, and the associated need for repeat treatment associated with EP in patients with sporadic and FAP AA, judicious timing and shared decision‐making with patients is required.

## CONFLICT OF INTEREST

Author: A.B.: Consultant: Boston Scientific, Steris, Medtronic Royalties: Medtronic. C.B.: Research: Ferring Pharmaceuticals, Janssen Pharmaceuticals, Cancer Prevention Pharmaceuticals, Freenome, Inc., Emtora Biosciences; Consultant: Freenome, Inc., SLA Pharma, Sebela Pharma; Speaker: Ambry Genetics. P.V.D.: Consultant for Olympus, Fujifilm, Boston Scientific, Microtech, Medtronic. D.L.: Consultant: Olympus. M.N.: Consultant: Boston Scientific, Olympus Amerika, Lumendi. V.K.: Consultant: Boston Scientific, Cook, Olympus, Medtronic; Research support: Allurion. S.N.: Consultant: Boston Scientific, Olympus, and Neptune Medical. N.S.: Consultant: Boston Scientific, Medtronic, STERIS, Olympus for educational and R&D. V.K.: Consultant for Cook Medical, Ambu, Steris, CDX, Motus GI, Castle Biosciences. The other authors declare no conflict of interest for this article.

## FUNDING INFORMATION

American College of Gastroenterology Medical Resident Clinical Research Award.

## Supporting information


**Table S1** Present literature on ampullary adenoma recurrence after endoscopic papillectomy in familial adenomatous polyposis and the sporadic population.
**Table S2** Univariate analysis of factors for recurrence among the entire cohort.
**Table S3** Characteristics of the recurrent ampullary adenomas.
**Figure S1** Kaplan–Meier curve for recurrence of ampullary adenomas after resection of the entire cohort; stars demonstrate occurrence of ampullary cancer at recurrence.
**Figure S2** Love plot for propensity score weighting.
