# Supplementary material for: Incidence and risk factors for recurrence of ampullary adenomas after endoscopic papillectomy: Comparative analysis of familial adenomatous polyposis and sporadic ampullary adenomas in an international multicenter cohort
Source: Dig Endosc. 2024 Jan 10;36(7):834–42. doi: 10.1111/den.14725 (PMC12136244; doi:10.1111/den.14725)
Supplement: Supplementary file 1 — Table S1 Present literature on ampullary adenoma recurrence after endoscopic papillectomy in familial adenomatous polyposis and the sporadic population. Table S2 Univariate analysis of factors for recurrence among the entire cohort. Table S3 Characteristics of the recurrent ampullary adenomas. Figure S1 Kaplan–Meier curve for recurrence of ampullary adenomas after resection of the entire cohort; stars demonstrate occurrence of ampullary cancer at recurrence. Figure S2 Love plot for propensity score weighting. [file DEN-36-834-s001.docx]

**Incidence and Risk Factors for Recurrence of Ampullary Adenomas after Endoscopic Papillectomy: A Comparative Analysis of FAP and Sporadic Ampullary Adenomas in an International Multicenter Cohort: Supplement**

| Supplement Table 1. Present literature on ampullary adenoma recurrence after endoscopic papillectomy in FAP and the sporadic population. | | | | | | | | | | |
| --- | --- | --- | --- | --- | --- | --- | --- | --- | --- | --- |
| **Author,**  **Year** | **Study Type** | **Countries** | **Patients**  **(N)** | **Sporadic AA** | **FAP AA** | **Follow-up** | **Recurrence** | | | **Risk factors for recurrence** |
|  |  |  |  |  |  |  | **Total** | **Sporadic** | **FAP** |  |
| Spadaccini,  2019 (8) | Systematic review | -* | 1751 | 1170 (66.8%) | 581 (33.2%) | 9.6-84.5 months | 11.80% | - | - | Not assessed |
| Ramai,  2021 (9) | Systematic review | -# | 99 | 0 | 99 | - | 25.40% | - | 25.40% | Not assessed |
| Kawashima,  2020 (12) | Single center, retrospective | Japan | 253 | 237 (93.7%) | 16 (6.3%) | 46 (0-192)  months | 16.90% | 19/199 (9.5%) | 5/13 (38.4%) | Female, FAP, intraductal extension |
| Roos,  2020 (10) | Single center, retrospective | Netherlands | 29 | 0 | 29 | 59 (12-108)  months | 52.2% | - | 12/23 (52.2%) | Not assessed |
| Fritzsche,  2021 (11) | Multicenter, retrospective | Australia, Netherlands | 154 | 111 (62.1%) | 43 (27.9%) | 40 (586 person-years) | 24 (15.6%) | 16/117 (13.6%) | 8/37 (23.5%) | No risk factors identified |
| Takahashi,  2021(13) | Retrospective study | Japan | 96 | 96 | 0 | 55 (6-216)  months | 13 (13.5%) | 13.5% | - | Positive non vertical margin on pathology, piecemeal resection |
| Foot note: Systematic review included *29 studies, # 5 studies  Abbreviation: AA: Ampullary adenoma, FAP: Familial adenomatous polyposis | | | | | | | | | | |

| Supplement Table 2. Univariate Analysis of factors for recurrence among the entire cohort | | | | |
| --- | --- | --- | --- | --- |
| **Factors** | **All**  **(N=257)** | **Hazard Ratio** | **95% CI** | **P-value** |
| Age | 60.0 [49.0;72.0] | 1.02 | [1.00, 1.03] | **0.03** |
| Race | 56 (22.0%) | 0.61 | [0.35, 1.05] | 0.07 |
| Male | 141 (55.1%) | 0.80 | [0.54, 1.18] | 0.26 |
| Ampullary adenoma Type: |  |  |  |  |
| Sporadic | 157 (61.1%) |  |  |  |
| Familial | 100 (38.9%) | 0.956 | [0.65, 1.41] | 0.82 |
| AA symptomatic: | 63 (24.8%) | 1.54 | [0.99, 2.40] | 0.05 |
| Pre-procedural imaging findings: |  |  |  |  |
| No abnormalities: | 77 (30.0%) | 0.45 | [0.28, 0.71] | **<0.01** |
| Biliary intraductal extension: | 9 (3.50%) | 0.93 | [0.34, 2.55] | 0.891 |
| Biliary Duct dilation: | 66 (25.7%) | 2.367 | [1.55, 3.62] | **<0.01** |
| Ampullary mass: | 68 (26.5%) | 1.222 | [0.76, 1.97] | 0.41 |
| Pancreatic Duct dilation: | 29 (11.3%) | 1.507 | [0.84, 2.70] | 0.17 |
| AA size at resection (mm) | 15.0 [10.0;20.0] | 1.025 | [1.01, 1.04] | **<0.01** |
| Post-papillectomy histology: |  |  |  |  |
| TA-LGD | 180 (70.0%) |  |  |  |
| TVA-LGD/VA-LGD | 36 (14.0%) | 1.487 | [0.84, 2.63] | 0.171 |
| HGD | 35 (13.6%) | 1.098 | [0.60, 2.02] | 0.76 |
| Papillectomy technique: |  |  |  |  |
| Endoscopic papillectomy alone | 120 (47.1%) |  |  |  |
| with submucosal injection | 135 (52.9%) | 0.803 | [0.542, 1.19] | 0.27 |
| Peri-ampullary involvement | 43 (20.9%) | 2.971 | [1.80, 4.89] | **<0.01** |
| Electrocautery settings: |  |  |  |  |
| Endocut | 114 (46.9%) |  |  |  |
| Coagulation | 21 (8.64%) | 1.570 | [0.76, 3.26] | 0.27 |
| Was the resection en-bloc or piecemeal?: | | | | |
| Piecemeal | 128 (51.0%) |  |  |  |
| Enbloc | 123 (49.0%) | 0.594 | [0.396, 0.89] | **0.012** |
| Adjuvant ablation therapy of the AA borders: | | | | |
| APC: | 82 (31.9%) | 1.423 | [0.957, 2.115] | 0.081 |
| Snare tip | 17 (6.61%) | 0.934 | [0.407, 2.145] | 0.873 |

| **Supplement Table 3.** Characteristics of the recurrent ampullary adenomas | | | | |
| --- | --- | --- | --- | --- |
| **Characteristic** | **Total**  **(N=106)** | **Sporadic**  **(N=58)** | **FAP**  **(N=48)** | **P-value** |
| Total recurrence: |  |  |  | <0.02 |
| Up to 1 year | 60 (28.3%) | 41 (50.6%) | 19 (43.8%) |  |
| 1-3 years | 28 (26.4%) | 8 (13.8%) | 20 (41.7%) |  |
| 3-5 years | 17 (16.0%) | 9 (15.5%) | 8 (16.7%) |  |
| >5 years | 1 (0.94%) | 0 (0.00%) | 1 (2.08%) |  |
| Site of recurrence: |  |  |  | 0.201 |
| Ampullary | 82 (78.8%) | 41 (73.2%) | 41 (85.4%) |  |
| Peri-ampullary | 22 (21.2%) | 15 (26.8%) | 7 (14.6%) |  |

**Supplement Figure 1.** Kaplan Meier curve for recurrence of ampullary adenomas after resection of the entire cohort, stars demonstrate occurrence of ampullary cancer at recurrence


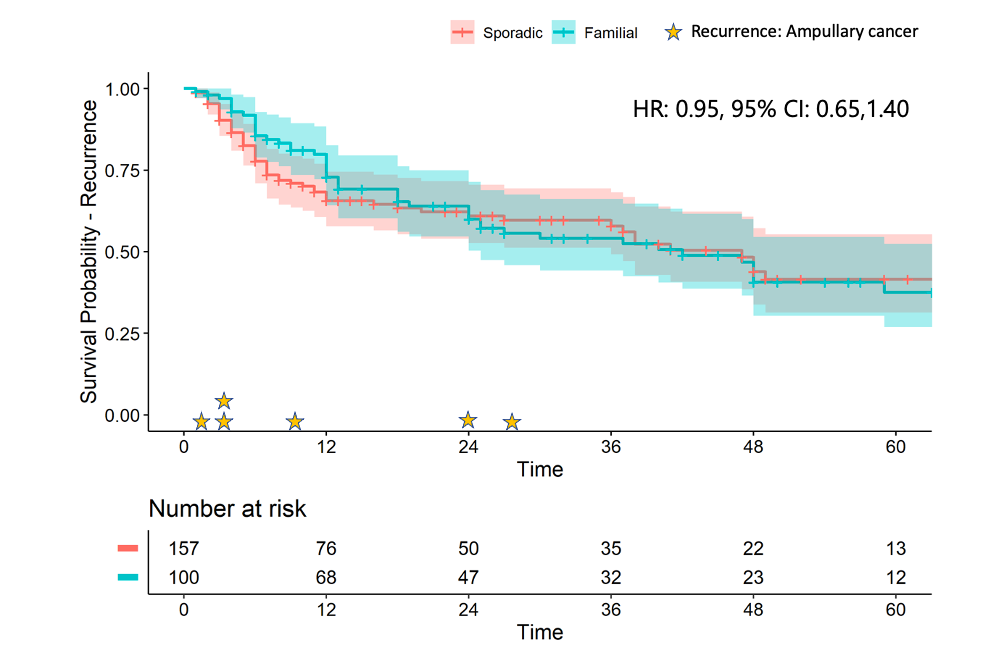


**Supplement Figure 2.** Love Plot for propensity score weighting

**
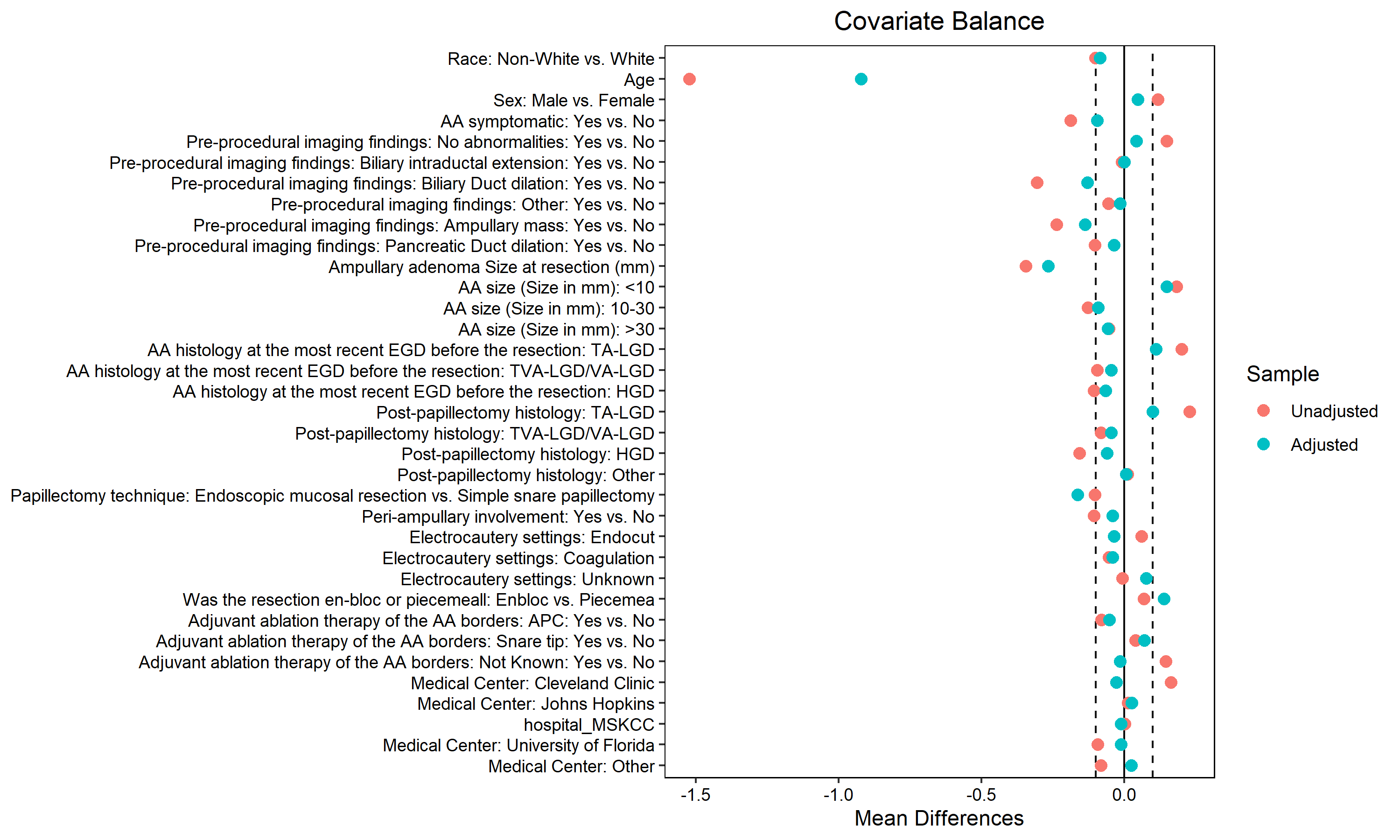
**
